# Structural Vaccinology for Viral Vaccine Design

**DOI:** 10.3389/fmicb.2019.00738

**Published:** 2019-04-16

**Authors:** Mohd Ishtiaq Anasir, Chit Laa Poh

**Affiliations:** Centre for Virus and Vaccine Research, Sunway University, Bandar Sunway, Malaysia

**Keywords:** structural vaccinology, vaccine design, protein structure, epitope, virus

## Abstract

Although vaccines have proven pivotal against arrays of infectious viral diseases, there are still no effective vaccines against many viruses. New structural insights into the viral envelope, protein conformation, and antigenic epitopes can guide the design of novel vaccines against challenging viruses such as human immunodeficiency virus (HIV), hepatitis C virus, enterovirus A71, and dengue virus. Recent studies demonstrated that applications of this structural information can solve some of the vaccine conundrums. This review focuses on recent advances in structure-based vaccine design, or structural vaccinology, for novel and innovative viral vaccine design.

## Introduction

Vaccination has considerably reduced morbidity and mortality caused by viruses such as smallpox virus and poliovirus. However, there remain many viruses without effective vaccines including dengue virus, human immunodeficiency virus (HIV), enterovirus A71, and hepatitis C virus. In addition, emerging infectious diseases will continue to threaten public health. Therefore, rapid vaccine development is mandatory to prevent pandemics. Currently, there are only 17 licensed vaccines in the United States against 13 viruses. These vaccines are either live-attenuated vaccines (11 vaccines), whole-inactivated vaccines (4 vaccines), or virus-like particle vaccines (2 vaccines) (Graham and Sullivan, 2018). Notably, the inactivated and live-attenuated vaccines are based on technologies developed decades ago. Taking live-attenuated vaccines as an example, the development of this type of vaccine is empirical and time-consuming. The virulent virus will be passaged in either tissue cultures or live animals until the avirulent form was isolated. In the settings of challenging viruses, these traditional approaches have failed to produce effective vaccines. Additionally, traditional approaches would not be swift enough to respond to epidemics caused by emerging viruses. This is due to lack of knowledge and experience with viral pathogenesis, viral growth, and attenuating determinants of the emerging viruses (Graham and Sullivan, 2018). Therefore, the focus for vaccine development against challenging viruses should be based on newer rational vaccine design platforms. In this article, we will cover recent advances in the development of novel vaccines facilitated by structural vaccinology. Additionally, we will discuss how structural vaccinology could lead to the discovery of antibodies for passive immunization. Finally, we will provide suggestions of how structural vaccinology can be exploited for future vaccine design.

## Structural Vaccinology Approach

**FIGURE 1:**
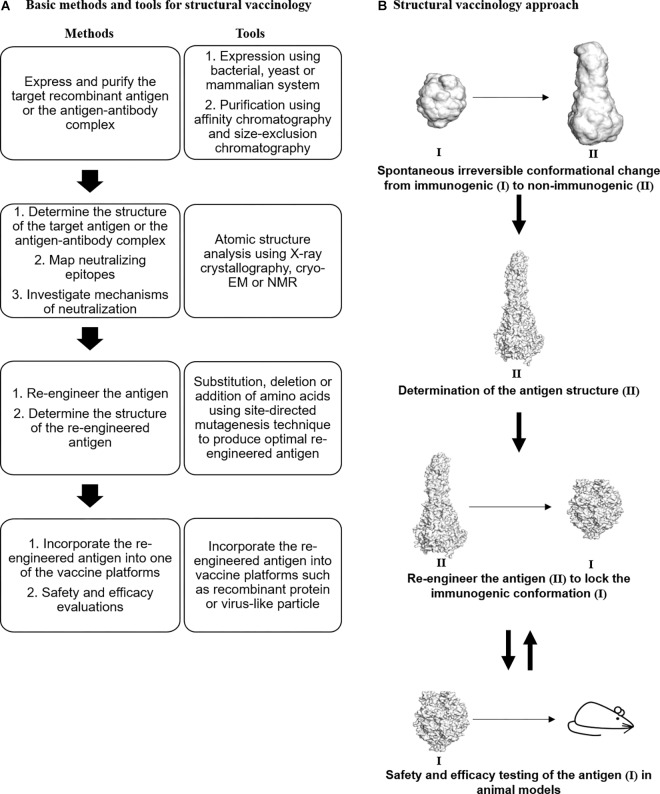
Structural vaccinology for vaccine design. **(A)** Basic methods and tools for structural vaccinology. **(B)** Irreversible conformational change (immunogenic to non-immunogenic) of viral antigens occurred spontaneously during recombinant antigen expression. To overcome this problem, the three-dimensional structure of the antigen (II) will be determined using structural biology techniques such as X-ray crystallography, cryo-electron microscopy (cryo-EM), and nuclear magnetic resonance (NMR). Based on the structure, the antigen will be re-engineered to produce an optimal antigen adopting a specific immunogenic conformation (I). Next, the re-engineered antigen could be incorporated into one of the vaccine platforms such as the recombinant protein vaccine platform. Thereafter, the safety and efficacy of the candidate vaccine should be tested in animal models. The antigen could be re-designed based on the animal testing evaluations to improve its immunogenicity and efficacy. RSV F glycoprotein was used as an illustrative example. PDB IDs: 4MMU and 3RKI.

Structural biology approaches have enabled the determination of the structure of whole viruses, viral proteins, and antigen–antibody complexes (Kaufmann et al., 2010; Lee and Wilson, 2015; Anasir et al., 2017a,b). The three-dimensional (3D) structures provide crucial insights into the tertiary structure and position of the viral epitopes. Ultimately, the detailed structural information can be exploited to solve some of the challenging problems impeding vaccine development against viruses. Structural vaccinology is a rational approach to generate an effective vaccine that involves (1) determining the atomic structure of the antigen or antigen–antibody complex, (2) remodeling the antigen or the epitope by reverse molecular engineering, (3) incorporating the re-engineered antigen or epitope into one of the vaccine platforms, and (4) testing the safety and efficacy of the candidate vaccine *in vivo* (Figure 1).

In the first step of this approach, the 3D structure of the antigen or antigen–antibody complex will be determined using either one or more of the structural biology tools such as X-ray crystallography, cryo-electron microscopy, and nuclear magnetic resonance. Thereafter, the antigen will be remodeled based on the structural information of the antigen. In addition, the immunological and bioinformatics knowledge should be synergistically combined with the structural insights to design an effective antigen. In most cases, the re-engineering process involves mutations of certain amino acids to stabilize immunogenic conformations, mask unwanted non-neutralizing antibody epitopes, optimize antigen thermostability, or introduce additional epitopes on the antigen. Next, the re-engineered antigen could be incorporated into one of the vaccine platforms such as the recombinant protein vaccine, the virus-like particle vaccine, or the live-attenuated vaccine. Finally, the immunogenicity and efficacy of the candidate vaccine should be tested in animal models including mice, ferrets, and macaques. Based on the preclinical findings, the candidate vaccine could be redesigned to improve its immunogenicity and efficacy. Structural vaccinology was shown to be successful in overcoming the challenges facing the bacterial vaccine field. For instance, the 3D structures of H-binding protein of Men B and the pili of group B *Streptococcus* have facilitated the design of improved vaccines (Van Regenmortel, 2016). Similarly, structural vaccinology has been used to improve the efficacy of viral vaccines.

## Human Immunodeficiency Virus

Human immunodeficiency virus is a serious public health threat with more than 35 million people living with HIV/AIDS worldwide in 2017. To date, there are no effective vaccines or drugs against HIV. Major barriers to vaccine development include the extreme diversity of HIV strains and the high malleability of its envelope. Due to these barriers, protective immune responses against HIV tend to be polyclonal and involve antibodies directed to a plethora of epitopes. Having structural information for the variable antigens in the viral envelopes is essential to design a vaccine that can elicit potent broadly neutralizing antibodies. HIV is one of the most studied pathogens in the context of structural vaccinology application. However, the development of an effective vaccine against HIV through a structural vaccinology approach remains elusive.

### Modification of Envelope Glycoprotein gp120

The glycoprotein (gp) 120 that is present on the surface of the HIV envelope has been identified as the main target for neutralizing antibodies (Pantophlet and Burton, 2006). However, its variability and malleable nature contribute to host immune evasion (Chen et al., 2009). Several groups have reported the development of structure-based vaccine candidates based on the modified gp120. For instance, structure-based modifications of gp120 significantly stabilized its core domain at the co-receptor binding site (Dey et al., 2009). Subsequent immunization studies revealed that the conformational stabilization was associated with increased humoral immune responses targeting the stabilized site. Although the initial modifications were unsuccessful to produce an effective vaccine against HIV, the study initiated the screening for broadly neutralizing antibodies against re-engineered gp120 proteins (Wu et al., 2010; Huang et al., 2018).

Another major barrier to HIV vaccine development is the induction of non-neutralizing antibodies by numerous epitopes on its envelope (McGuire et al., 2014). Such antibodies could hinder the function of its broadly neutralizing counterparts. The V3 loop of gp120 is an example of a non-neutralizing antibody epitope. Previous studies revealed that V3 immunogenicity could be diminished by introducing a hydrophobic amino acid substitution (A316W mutation) to reduce its conformational flexibility (de Taeye et al., 2015). Recently, two hydrophobic mutations, S306L and R308L, were found to further stabilize the V3 region, potentially by maintaining the burial of the V3 region in the pocket formed by V1/V2 domains (de Taeye et al., 2018). Binding studies showed that these hydrophobic mutations impaired non-neutralizing antibody interactions with the V3 loop. Immunization studies in rabbits revealed that the stabilization of the V3 region was associated with reduced induction of non-neutralizing antibodies, while the induction of neutralizing antibodies remained the same. These data demonstrated that structural vaccinology could be exploited to reduce the induction of non-neutralizing antibodies.

### Stabilization of Prefusion-Closed HIV Envelope

Human immunodeficiency virus envelope in its prefusion-closed state is the main target of broadly neutralizing antibodies (Wang et al., 2016; Ozorowski et al., 2017). Due to the extreme diversity of HIV strains, sequential immunization with HIV envelopes adopting the prefusion-closed conformation from different strains might be needed to induce sufficiently broadly neutralizing antibodies. Despite many attempts to stabilize the HIV envelope in its prefusion-closed state, these variants were found to fluctuate between closed and open prefusion states (Ozorowski et al., 2017). Recently, Rutten et al. (2018) described a universal approach to produce high-quality prefusion-closed HIV envelopes through a repair-and-stabilize approach (Rutten et al., 2018). In the repair stage, they substituted rare strain-specific residues of the envelopes with more prevalent residues. In the stabilization stage, regions crucial for envelope refolding were modified to increase stability. One of the variants designated as ConC_base0, which carried seven stabilizing substitutions, was found to be stable at 37°C for 4 weeks. This variant exhibited increased binding toward broadly neutralizing antibodies along with reduced affinity toward non-neutralizing antibodies. Detailed inspection of the variant structure revealed that the inter- and intra-protomer stabilizing mutations provided synergistic effect to stabilize the envelope in the prefusion-closed structure. Hence, this approach may be a better strategy to generate high-quality prefusion-closed HIV envelopes for future development of efficacious vaccines against HIV.

### Monoclonal Antibodies for Passive Immunization Against HIV

The screening of broadly neutralizing antibodies against HIV was initiated by the structural determination of the gp120–CD4 receptor complex (Kwong et al., 1998). Earlier, the attempts to generate broadly neutralizing antibodies specifically directed toward the CD4 binding site were unsuccessful. The failure was partly due to the fact that the gp120 used in the screening was reactive with many other antibodies, including the non-neutralizing antibodies. To generate specific antibodies targeting the CD4 binding site, gp120 was modified to eliminate the other antigenic regions while preserving the CD4 binding site (Wu et al., 2010). One of the variants designated as resurfaced stabilized core 3 (RSC3) was chosen to be used in the screening of broadly neutralizing monoclonal antibodies. From the screening, three monoclonal antibodies, which are VRC01, VRC02, and VRC03, were found to bind strongly and specifically to RSC3. VRC01 displayed impressive breadth of reactivity by neutralizing 91% of the 190 circulating HIV-1 genetic subtypes. Subsequent structural studies revealed that VRC01 engaged the CD4 binding site of gp120 in a similar manner to the CD4–gp120 interactions (Zhou et al., 2010). Currently, VRC01 is in the phase II clinical trial as a passive immunization strategy (Huang et al., 2018).

## Hepatitis C Virus

Hepatitis C virus is the leading cause of liver diseases such as cirrhosis, liver failure, and liver cancer. More than 64 million people are chronically infected with hepatitis C virus (Manns et al., 2017). Liver transplantation is the main treatment undertaken for those with cirrhosis and liver cancer (Manns et al., 2017). However, the treatment does not fully eliminate the virus, resulting in possible viral rebound and infection of the transplanted liver (Manns et al., 2017). High cost is also associated with other antiviral treatments (Cox, 2015), underscoring the importance of developing a vaccine against hepatitis C virus.

### Epitope-Based Vaccines Against Hepatitis C Virus

Similar to HIV, the development of a vaccine against hepatitis C virus has been hampered by the variability and diversity of target antigens. E2 glycoprotein is the main target for hepatitis C vaccine development. To date, the structure of full-length E2 has yet to be determined (Pierce et al., 2017). Importantly, two crystallographic structures of the engineered E2 core from two genotypes are available (Kong et al., 2013; Khan et al., 2014). In addition, various structures of hepatitis C viral antigens in complexes with broadly neutralizing antibodies have been determined (Kong et al., 2012a,b, 2015; Deng et al., 2013; Krey et al., 2013; Meola et al., 2015). These studies identified multiple epitopes that could elicit broadly neutralizing antibodies. One of the epitopes, designated as epitope I, is located near the N-terminus of E2. The epitope was found to elicit arrays of human (Keck et al., 2013) and murine antibodies (Tarr et al., 2006). Structural studies revealed that epitope I adopted a β-hairpin fold when bound to neutralizing antibodies (Kong et al., 2012b; Sandomenico et al., 2016).

Initially, a cyclic variant of epitope I adopting the β-hairpin fold was designed to generate and characterize monoclonal antibodies specific against the epitope variant (Sandomenico et al., 2016). This antigen was able to elicit antibodies in mice. However, the antibodies lacked neutralization activity when tested. Subsequently, Pierce et al. (2017) designed cyclic antigens to stably present epitope I in its antibody-bound conformation using the natural scaffold approach (Pierce et al., 2017). They identified θ-defensin protein, which adopted the β-hairpin substructure nearly identical to the antibody-bound epitope I structure (Conibear et al., 2013). Based on the cyclic θ-defensin structure, they designed two cyclic epitope I antigens comprising minimal scaffolded structures of the epitope. Immunogenicity studies revealed that the cyclic antigens induced higher epitope-specific serum responses in comparison to the linear peptide. Subsequent viral neutralization studies indicated that the cyclic antigens were superior immunogens than the linear epitope. However, the antibody responses elicited by the cyclic antigens were still low. Various options are available to improve cyclic epitope 1 immunogenicity. For instance, the cyclic antigens could be displayed on a virus-like particle or other nanoparticles to enhance immune responses against them.

In another approach, Pierce et al. (2017) designed a bivalent truncated E2 antigen displaying two copies of epitope I on its surface. By analyzing E2 core structures, they identified a site suitable to display the second epitope I. However, the bivalent antigen elicited lower neutralizing antibody responses than its monovalent counterpart. This indicated that further understanding of the host humoral responses against E2 is required for successful vaccine design in the future.

## Respiratory Syncytial Virus

Respiratory syncytial virus (RSV) is an etiological agent that causes lower respiratory tract infection in infants and children and often leads to hospitalizations. Previously, a formaldehyde-inactivated RSV vaccine candidate was found to enhance the disease upon subsequent infection with the wild-type virus, while live-attenuated vaccine development has yet to unearth an acceptable balance of RSV attenuation (Moghaddam et al., 2006).

### Prefusion F Stabilization

**FIGURE 2:**
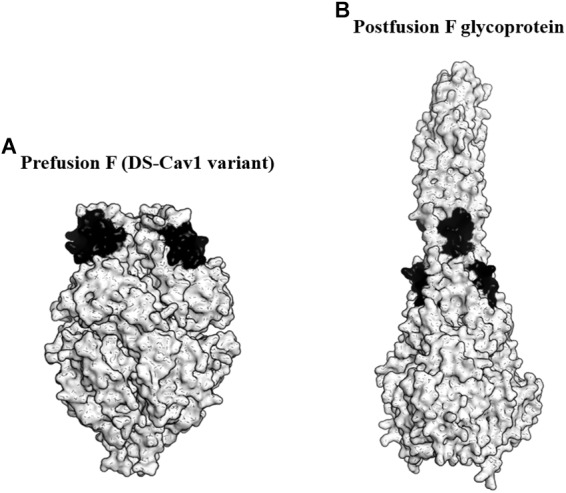
Targeting the prefusion F glycoprotein of respiratory syncytial virus (RSV). **(A)** Surface representation of the DS-Cav1 F glycoprotein variant (PDB ID: 4MMU). DS-Cav1 adopted the prefusion F glycoprotein conformation. The antigenic site ø is shown in black. **(B)** Surface representation of the trimeric postfusion F glycoprotein (PDB ID: 3RKI). The disrupted antigenic site ø caused by structural rearrangement from the prefusion to postfusion F is shown in black.

F glycoprotein is an attractive antigen for vaccine development as it contains multiple neutralizing epitopes. The protein forms a lollipop-shaped structure with its C-terminus inserted into the virion membrane. During viral entry, it rearranges from a metastable prefusion state to a highly stable postfusion state (Figure 2). Previous studies revealed that the metastable prefusion state accounted for most of the neutralizing activity (Magro et al., 2012). Moreover, McLellan et al. (2013b) identified an antigenic site ø that was bound by several prefusion F-specific antibodies. The antigenic site ø is located at the top of the prefusion F trimer and is readily accessible by the antibodies even on the crowded virion surface. The prefusion F was also found to be the target of RSV-neutralizing activity in human sera. Importantly, the antigenic site ø is only present on prefusion F and not on the postfusion F antigen (Steff et al., 2017).

To identify the prefusion F with the highest stability at the antigenic site ø, McLellan et al. (2013a) produced over 100 variants of the protein. These variants possessed mutations either to stabilize the antigenic site ø or to destabilize the postfusion F. From the study, they identified a variant favoring the prefusion conformational state designated as DS-Cav1. DS-Cav1 carried S155C-S209C double mutations to form a disulfide bond and cavity-filling mutations (S190F and V207L). Studies in mice, macaques, and calves revealed that DS-Cav1 elicited potent neutralizing responses (McLellan et al., 2013a; Steff et al., 2017). Crucially, the studies using calves concluded that the use of DS-Cav1 was able to extend the duration of protection conferred by maternal immunization, potentially lowering RSV disease burden in infants (Steff et al., 2017). In 2017, DS-Cav1 entered phase I clinical trial and the trial is currently ongoing.

## Influenza A Virus

Influenza causes significant disease and death in the human population especially among the young and elderly (Shao et al., 2017). Currently, individuals are vaccinated with seasonal influenza vaccine that stimulates humoral immune responses toward the globular head domain of the viral hemagglutinin (HA). HA is a glycoprotein that acts as a mediator of viral entry into host cells (Sautto et al., 2018). One major problem with the current vaccine is that the antibodies elicited are highly strain specific, as the globular head domain is not conserved across strains. Therefore, the vaccines must be reformulated annually based on the anticipated influenza strain. Furthermore, most influenza vaccines are produced in an egg-based production system. Previous study showed that this production system caused amino acid substitutions on the HA during passaging and led to reduction in vaccine efficacy (Wu et al., 2017). In addition, the production process is slow and can cause severe production bottleneck during an epidemic.

### Universal Influenza Vaccine

Many investigators characterized antibody–HA complexes to elucidate the host humoral immune responses against influenza A. Corti et al. (2011) identified a broad neutralizing antibody targeting the F subdomain within the highly conserved stem domain of HA. Subsequent comprehensive sequence analysis revealed a high degree of conservation within the stem domain, unlike the variable head domain (Mallajosyula et al., 2015). More recently, evolutionary analysis indicated that the stem domain evolved slowly over time than the evolution of the head domain (Kirkpatrick et al., 2018). In addition, the study found that the stem evolution did not aid in the host immune evasion. Thus, universal vaccine development against influenza virus strains should focus on the HA stem region instead of the head region.

To develop a universal influenza vaccine, Krammer et al. (2013) designed multiple chimeric HA (cHA) constructs combining the stem domain from the H1 strain with the unique head domain derived from various strains. They tested the protective potential of the cHA constructs against multiple challenge viruses (Hai et al., 2012; Krammer et al., 2013). Sequential vaccination with the cHA constructs in mice elicited stem-specific polyclonal humoral responses. The vaccination also protected mice against heterologous and heterosubtypic influenza virus challenges (Hai et al., 2012). Subsequent studies using female ferrets revealed that the vaccination with cHA reduced viral titers in nasal turbinates, lungs, and olfactory bulbs (Krammer et al., 2014). Recently, a confirmatory study was performed in male ferrets. It was found that live-attenuated vaccines expressing cHAs were indeed able to confer protection during virus challenge (Nachbagauer et al., 2018).

In a second approach, another group designed immunogens comprising only the stem region designated as mini-HAs (Impagliazzo et al., 2015). They constructed multiple mini-HAs that were stable as either monomers or trimers. The mini-HAs were found to induce high titers of antibodies binding to the HA of group 1 (H1, H5, and H9) and group 2 (H3 and H7) influenza A strains. The trimeric mini-HAs displayed full protection of mice against heterosubtypic influenza challenge. In contrast, the monomeric mini-HAs only conferred partial protection. Consistently, monkeys vaccinated with the trimeric mini-HA had significantly reduced fever after being challenged with the H1N1 virus. Many influenza vaccines based on the cHA and headless HA approaches are currently undergoing clinical trials (WHO). For instance, in an ongoing phase I clinical trial, adults aged from 18 to 39 were given a live-attenuated influenza vaccine expressing cHA followed by an inactivated vaccine expressing another cHA (NIH, United States).

### Multidomain Antibody to Confer Protection Against Influenza Infection

Due to the conservation of the HA stem region, many groups are actively developing antibodies directed toward the stem domain (Krammer et al., 2014; He et al., 2015). The stem-specific antibodies displayed broader specificities than the antibodies directed toward the HA head domain (Joyce et al., 2016). Although several of these antibodies have entered clinical trials, their use as prophylactic agents remained elusive due to incomplete coverage of the various influenza strains.

Laursen et al. (2018) utilized structure-based antibody design with the aim to develop an antibody that could provide broad and long-lasting protection against influenza A and B strains. They designed a multidomain antibody designated as MD3606 comprising four antibodies linked to a human IgG1 Fc. The four antibodies were SD36, SD38, SD83, and SD84. SD36 was found to neutralize influenza A virus group 2, while SD38 was found to neutralize both group 1 and group 2. Additionally, SD84 and SD83 were found to neutralize all influenza B strains. Structural analysis of these antibodies revealed that these antibodies were able to bind to highly conserved epitopes. When administered intravenously or expressed locally by a viral vector, MD3606 was found to confer broad protection in mice against influenza A and B viral infections. Further development of MD3606 is warranted as it has the potential to confer passive protection throughout the influenza season, provided the preclinical findings could be replicated in clinical trials.

## Dengue Virus

In recent decades, dengue virus (DENV) has emerged as a significant public health threat with an estimated 390 million infections annually (Bhatt et al., 2013). Dengue infections are caused by four antigenically distinct serotypes (DENV1-4) that can cross-react with each other immunologically. Currently, there is only one recombinant live-attenuated vaccine (Dengvaxia) approved for dengue prevention in endemic populations (Guy et al., 2015). However, the vaccine has low efficacy (53%) for seronegative individuals (Hadinegoro et al., 2015). Dengvaxia is also associated with increased risk of hospitalization for dengue among vaccinated children from 2 to 5 years of age (Hadinegoro et al., 2015). A recent clinical trial concluded that Dengvaxia led to increased risk of antibody-dependent enhancement (ADE) in the seronegative individuals (Halstead, 2017). ADE is a phenomenon in which the elicited antibodies during primary infection are cross-reactive with the heterologous serotypes but are non-neutralizing. The antibody-bound virus is more easily internalized by Fcγ receptor-positive myeloid cells, leading to infection enhancement. Thus, it is desirable for a vaccine to induce only neutralizing antibodies but not non-neutralizing antibodies. In the case of dengue infection, ADE has been identified as the central mechanism associated with dengue hemorrhagic fever and dengue shock syndrome during secondary infections (Guzman et al., 2013). Thus, the success of dengue vaccine design hinges on the elimination of ADE in addition to balanced immune responses against all four serotypes.

### Masking of Non-neutralizing Epitopes

**FIGURE 3:**
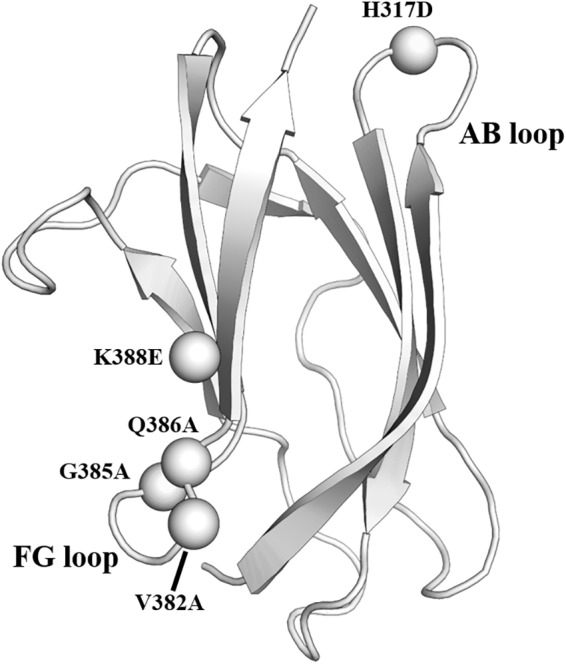
Resurfaced domain III of the envelope glycoprotein. Cartoon representation of domain III of DENV2 envelope glycoprotein. The residues in the FG loop and AB loop that were mutated in the rsDIII-Ala30 are shown as spheres and labeled. PDB ID: 1OAN.

The mature virion of DENV is coated by glycoprotein E. It contains three domains, which are domain I (EDI), domain II (EDII), and domain III (EDIII) (Kuhn et al., 2002). EDIII is an attractive antigen as it is targeted by broadly neutralizing antibodies. However, previous animal studies revealed that although immunization using EDIII proteins induced robust antibody responses, neutralization was modest and limited to serotype-specific responses (Hermida et al., 2006; Saejung et al., 2007; Zhang et al., 2007; Batra et al., 2010). The presence of non-neutralizing antibodies targeting the immunodominant epitopes likely accounted for the limited neutralization potential of EDIII protein (Frei et al., 2018). To address the issue, a panel of resurfaced EDIII proteins was designed. Non-neutralizing epitopes such as the AB loop epitope and the serotype-specific FG loop epitope were masked in these variants (Frei et al., 2018). One of the resurfaced EDIII designated as rsDIII-Ala30 was found to only bind to the neutralizing antibodies 4E11 and 4E5A, but not the non-neutralizing antibody (2H12) and the serotype-specific antibody (3H5) (Figure 3). Upon immunization in mice, rsDIII-Ala30 elicited broadly reactive and cross-neutralizing antibody responses against DENV1-3. However, its antisera are insufficient to confer protection from DENV2 infection in mice.

### Structure-Based Anti-dengue Antibody Design

Previously, antibody 4E11 was identified to neutralize dengue viruses of all serotypes (Thullier et al., 1999, 2001; Cockburn et al., 2012). However, its neutralizing activity was limited, potentially due to epitope inaccessibility on the mature virion (Tharakaraman et al., 2013). To improve the neutralizing potential of 4E11, Tharakaraman et al. (2013) produced a 4E11 variant designated as 4E5A that carried five amino acid substitutions. 4E5A showed marked improvement in affinity toward EDIII of DENV4. However, the affinity of 4E5A toward DENV4 is modest in comparison to its affinities to other DENV serotypes. Subsequently, they performed structural analysis to design an antibody designated as Ab513 (Robinson et al., 2015). This antibody differed from the starting murine antibody 4E11 through (1) six point mutations and deletion at position 26 to improve affinity toward DENVs and (2) mutations to humanize the antibody. Challenge studies revealed that the mice administered with Ab513 had accelerated recovery of platelet level post-DENV challenge. Furthermore, the passively immunized mice displayed significant reduction in the viral load upon challenge. Currently, Ab513 is in the preclinical trial to determine the optimal dosing scheme for use as a therapeutic or prophylactic antibody against DENV infection.

## Enteroviruses Causing Hand, Foot, and Mouth Disease

Hand, foot, and mouth disease (HFMD) is a significant public health threat. In 2017, China experienced large-scale HFMD outbreaks of over 1.95 million cases with 96 fatalities (WHO Western Pacific). Besides China, HFMD outbreaks have been occurring periodically throughout the world in countries such as Taiwan, Japan, Vietnam, Singapore, and Malaysia. The main etiological agents for HFMD are EV-A71 and CV-A16. Currently, there are three licensed formaldehyde-inactivated HFMD vaccines in China. However, these vaccines could only confer protection at 80% efficacy against severe HFMD in comparison to 95% efficacy against mild HFMD. In addition, the monovalent EV-A71 vaccine is not protective against HFMD caused by CV-A16. With the rising concern of HFMD outbreaks, there is a need for an effective bivalent vaccine against both EV-A71 and CV-A16.

### Bivalent Hand, Foot, and Mouth Disease Vaccine

**FIGURE 4:**
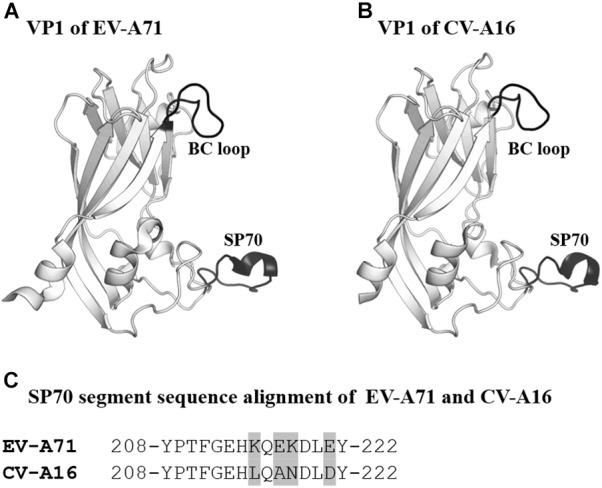
VP1 of EV-A71 and CV-A16. **(A,B)** Cartoon representation of VP1 (amino acids 76–276) of EV-A71 (PDB ID: 3VBS) and CV-A16 (PDB ID: 5C4W). The BC loop and SP70 region are shown in black and labeled. **(C)** Sequence alignment of SP70 segments of EV-A71 and CV-A16. Amino acids that are not conserved between the two viruses are highlighted in light gray.

The SP70 epitope is the main neutralizing epitope in EV-A71. The epitope was discovered to elicit neutralizing antibodies and conferred protection against homologous and heterologous EV-A71 strain challenges in neonatal mice (Foo et al., 2007a,b). The SP70 epitope lies in the GH loop of VP1 (aa208–222) situated at the surface of the viral capsid (Plevka et al., 2012; Lyu et al., 2015a,b). CV-A16 also possesses an amino acid segment corresponding to the SP70 epitope (Figure 4; Ren et al., 2013, 2015).

To develop a bivalent vaccine, Zhao et al. (2015) designed a chimeric EV-A71 Virus-Like Particle (ChiEV-A71 VLP). The SP70 peptide of EV-A71 was replaced with the corresponding SP70 sequence from CV-A16 in ChiEV-A71 VLP (Zhao et al., 2015). The VLP was found to elicit robust Th1/Th2-dependent immune responses against EV-A71 and CV-A16. In addition, passive immunization with sera raised against ChiEV-A71 VLPs conferred full protection against lethal challenge with EV-A71 and CV-A16 in neonatal mice. Structural studies revealed that the SP70 epitope replacement converted the VLP surface charge potential from negative to neutral (Lyu et al., 2015a). The shift in the surface charge potential coupled with variations in amino acid sequences most likely accounted for the additional neutralization capability of the ChiEV-A71 VLP. Apart from EV-A71 and CV-A16, other coxsackieviruses such as CV-A6 and CV-A10 are emerging HFMD etiological agents (Chen et al., 2017; Anh et al., 2018). Incorporation of the neutralizing epitopes of each virus into a single VLP will enable the construction of a multivalent HFMD vaccine, provided the right approaches including structural vaccinology were utilized during vaccine design.

### Improvement of Vaccine Thermostability

Despite being one of the most successful medical tools, there are still more than 17 million vaccine-preventable deaths caused by underutilization of vaccines (Clemens et al., 2010; Chen et al., 2011). This situation is especially true for the poorer countries, where cold chain is difficult to maintain, as most vaccines are sensitive to heat. The heat-unstable vaccines have poor efficacy when they are not properly refrigerated during delivery and storage. This led to incomplete immunizations during the implementation of vaccine programs (Schlehuber et al., 2011).

Self-biomineralization is one of the possible strategies to improve vaccine stability. Biomineralization is a mechanism where living organisms form exterior shells made of minerals such as calcium phosphate and calcium carbonate to cope with the harsh environments (Palmer et al., 2008; Farooq and Dietz, 2015). For instance, egg cells are enclosed by mineral shells to protect themselves from the external environment. Initially, research groups demonstrated that self-biomineralization could be mimicked through the incorporation of self-biomineralizing peptides (Hartgerink et al., 2001; Lee et al., 2009). Subsequently, Wang et al. (2013) incorporated self-biomineralizing peptides into the BC loop of attenuated EV-A71 to improve the thermostability of the candidate vaccine (Wang et al., 2013). From 3D structures of EV-A71, they identified that the BC loop was externalized from the capsid and was suitable to be the region for peptide insertion (Figure 4). Importantly, the attenuated EV-A71 incorporated with self-biomineralizing peptides displayed biomineralization capacity to form 5- to 10-nm exterior shells made of calcium phosphate. The vaccine exhibited enhanced thermostability, as it could be stored at 26°C for more than 9 days and at 37°C for 1 week. Intriguingly, they found that the biomineralized vaccine had improved immunogenicity, indicating an additional advantage of this approach.

## Structural Vaccinology Pitfalls

Structural vaccinology has a great potential, but it faces some limitations. Firstly, this technology is susceptible to incomplete structural and immunological knowledge of the virus-induced host immune responses. For instance, mapping of a dengue epitope on available dengue structures revealed that the epitope was inaccessible in any of the known dengue configurations (Li et al., 2018). This indicated that the antibody recognized an as-yet unresolved conformation of the E protein displaying the epitope on the dengue surface. Another drawback is the static nature of X-ray crystallographic analysis of the antigen–antibody complexes. X-ray crystallography is one of the most utilized structural biology technique to understand antigen–antibody complexes (Toride and Brooks, 2018). However, the static picture provided by X-ray crystallography tends to obscure the role of structural plasticity in facilitating antigen–antibody interactions (Van Regenmortel, 2016). Generally, the structures visualized in antigen–antibody complexes and the free molecules differed at the binding sites due to structural alterations during binding. Therefore, the structure of an antigen–antibody complex determined using X-ray crystallography does not necessarily equate to the actual immunogenic structure recognized by the B cell receptors during antibody production. Intriguingly, it can be noted that most of the structural vaccinology efforts attempted to improve the B-cell antibody responses, but not the T-cell responses. This is problematic as the T-cell response is known to be associated with many antiviral properties including viral clearance and decreased viremia (Rosendahl et al., 2014). Furthermore, induction of T-cell response negated the effect of viral mutations, ultimately preventing them from escaping the immune responses (Rosendahl et al., 2014).

In addition to the aforementioned pitfalls, there are several review papers that have discussed the limitations of structural vaccinology to develop an effective vaccine against HIV (Pejchal and Wilson, 2010; Van Regenmortel, 2016). These pitfalls underscore the importance of acquiring new structural and functional insights into humoral immune responses against pathogens. Furthermore, incorporation of multiple structural techniques especially nuclear magnetic resonance (NMR) is favorable to study the structure and molecular dynamics of antigen–antibody complexes (Moraes et al., 2016). As structural vaccinology is still developing, its application is primarily limited to the design of recombinant protein vaccine, peptide vaccine, and VLPs. These vaccine platforms are associated with low immunogenicity. Therefore, it is crucial to integrate structural vaccinology with other complementary key technologies. For instance, DS-Cav1 protein vaccine could be integrated with the single-round replication recombinant vesicular stomatitis virus vector to potentially enhance its immunogenicity if required (Johnson et al., 2013). Lastly, a structural vaccinology approach should be extended to improve T-cell responses and not just focus on the B-cell responses to produce better viral vaccines in the future.

## Conclusion

Many of the structure-based vaccine candidates are under development, and some of them are in clinical trials (Robinson et al., 2015; Sastry et al., 2017; Huang et al., 2018). Recent advances have proven that structure-based vaccines are feasible. In some cases, structural knowledge is mandatory to circumvent the problems faced by conventional strategies. Based on current evidence, structural knowledge is essential to stabilize malleable antigens. It also allows the modifications of proteins or envelopes, either to enhance antigenicity or mask the non-neutralizing epitopes (Dey et al., 2009; de Taeye et al., 2018; Frei et al., 2018). Moreover, structure-based modifications are useful to lock antigenic proteins or envelopes in a certain conformation to maximize epitope presentation (McLellan et al., 2013a; Rutten et al., 2018). In addition, structural vaccinology can be utilized to design antibody therapies (Robinson et al., 2015; Huang et al., 2018). Lastly, a structural vaccinology approach can be used to improve vaccine thermostability, potentially solving the cold-chain problem faced in remote and poorer areas in large continents (Wang et al., 2013).

## Author Contributions

MIA wrote the manuscript. CLP provided the critical revision and final approval of the manuscript.

## Conflict of Interest Statement

The authors declare that the research was conducted in the absence of any commercial or financial relationships that could be construed as a potential conflict of interest.
